# Clonal replacement of novel T cells: a new phenomenon in the tumor microenvironment following PD-1 blockade

**DOI:** 10.1038/s41392-019-0077-2

**Published:** 2019-10-25

**Authors:** Xue Li, Manni Wang, Rong Xiang

**Affiliations:** 1 Department of Biotherapy, State Key Laboratory of Biotherapy and Cancer Center, West China Hospital, Sichuan University, No. 17, Block 3, Southern Renmin Road, 610041 Chengdu, Sichuan China; 2 Department of Immunology, School of Medicine, Nankai University, Tianjin, China

**Keywords:** Cancer microenvironment, Cancer therapy

**Fig. 1:**
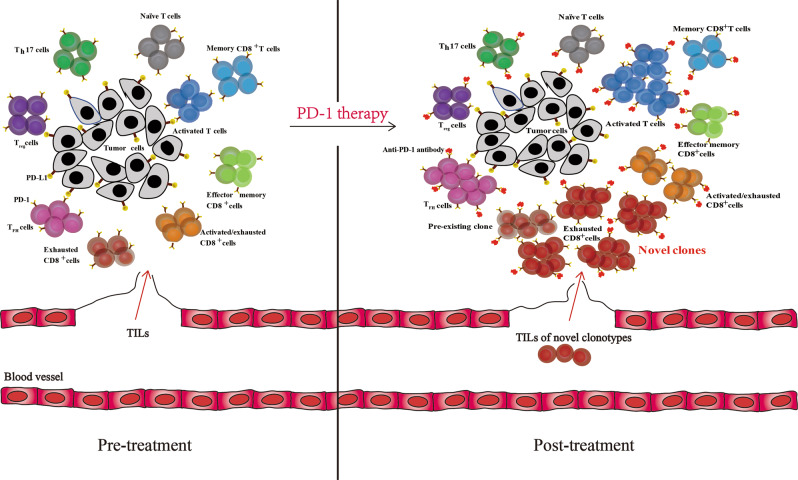
The pattern of the clonal replacement of tumor-specific T cells in the tumor microenvironment following PD-1 blockade

In a recent study published in Nature Medicine, Yost et al.^1^ described the clonal replacement of tumor-specific T cells after treatment with PD-1 antibodies. This finding reveals the intrinsic ability of the tumor microenvironment to attract new T cells, which has crucial applications for the design of immune checkpoint blockade (ICB) therapies (Fig. 1).

Over the past few decades, ICB therapies have been successful in some patients with different types of cancer by blocking inhibitory checkpoint receptors on T cells, and thus restoring T cell-mediated immune responses as well as recruiting more T cells into the tumor environment. It should be noted that the heterogeneity of the tumor microenvironment could lead to different responses to ICBs in cancer patients. However, the composition and origins of tumor-infiltrating lymphocytes (TILs) that have the actual antitumor activity after treatment with ICB therapies remain unclear.

The highly heterogeneous CD8+ TILs in lung and colorectal cancer tissues were previously believed to recognize not only tumor-specific antigens but also various epitopes that were unrelated to cancer.^2^ A recent publication in Nature Medicine, however, uncovered that only 10% of intratumoral CD8+ T cells were able to recognize autologous tumors. Moreover, a tumor-reactive T cell receptor (TCR) was not identified, despite the significant infiltration of T cells in patient tumor samples.^3^ Given the complexity of TILs, it is important to further investigate their diversity and origins for the purposes of both basic research and clinical applications.

Based on the results of single-cell RNA-sequencing (scRNA-seq) and TCR-sequencing (TCR-seq) of advanced basal cell carcinoma (BCC) patients pre-PD-1 and post-PD-1 therapies, Yost and his team identified 19 cell clusters, which included two malignant clusters with significant heterogeneity and other non-malignant clusters. The team then identified 9 distinct T cell clusters after reclustering 33,106 TILs and found that the exhausted CD8+ TIL population increased clonally following PD-1 treatment. These exhausted CD8+ TILs were identified by the expression of gene signatures involved in chronic activation, T cell dysfunction and tumor reactivity. The coexpression of CD39 (which is encoded by ENTPD1) and CD103 (which is encoded by ITGAE) is the hallmark of tumor-specific CD8+ T cells.^4^ It was also suggested that clonally expanded TILs were highly correlated in cellular phenotype that was unaffected by PD-1 blockade based on the clustering analysis of TCR sequences that showed that there were shared motifs in the CDR3 sequence among these TILs. Furthermore, the authors investigated whether the clonal replacement after PD-1 blockade relied on the remodeling of pre-existing TILs or on the recruitment of novel T cells. Comparing TRB (which encodes TCRβ) frequencies in the pretreatment and posttreatment BCC samples, the exhausted clones mainly consisted of novel clonotypes (~84% in each patient). Sixty-four percent (7/11) of patients presented an increased number of exhausted CD8+ T cell clones after treatment, the majority (6/7) of which were novel clonotypes. The results of bulk TCR-seq were consistent with scTCR-seq analysis.

It has been hypothesized that the expanded TILs of new clone types were recruited from sites outside of the tumor, such as lymphatic tissues or the tumor periphery. The researchers further found that 41% of TIL TRB clonotypes were detected in the blood samples of five patients through bulk TCR-seq analysis. At the same time, the proportion of exhausted T cells with new clonotypes found in the peripheral blood increased from 11.8% to 35.5% following PD-1 treatment. Additionally, in the scRNA-seq and scTCR-seq profiles of squamous cell carcinoma (SCC) tissues, similar patterns of clonal substitution of exhausted CD8+ T cells were observed. This finding suggests that the phenomenon of clonal replacement might be universal and may occur in different cancer types following anti-PD-1 treatment.

Previous studies have proposed that T cell dysfunction or exhaustion plays an important role in immunosuppression in tumor-bearing patients and can serve as a potential target for immunotherapies.^5,6^ However, the underlying changes in TILs, especially in exhausted T cells, are unknown. This study provides new insights into the clonal replacement of TILs in response to ICBs, which frequently results in the replacement of exhausted CD8+ T cells by novel clones that are potentially derived from the peripheral blood. This observation could be helpful for predicting the response of tumors to PD-1 blockade and for optimizing the design of checkpoint blockade immunotherapies. More efforts are needed to determine the specific origin and actual function of the novel T cell clones in the microenvironment and in clinical response. The potential mechanisms of clonal replacement in the tumor microenvironment are also worthy of investigation. Thus, future immunotherapies could focus on improving the intrinsic ability of tumors to attract more novel T cells from distant sites to boost the local antitumor immune responses. Another interesting topic that should be investigated is whether this phenomenon can be observed in other immune checkpoint therapies, such as cytotoxic T lymphocyte-associated antigen-4 (CTLA-4) blockade.
